# (Imidazole-κ*N* ^3^){*N*-[1-(2-oxidophenyl)ethylidene]-l-valinato-κ^3^ *O*,*N*,*O*′}copper(II)

**DOI:** 10.1107/S1600536808028134

**Published:** 2008-09-06

**Authors:** Gan-Qing Zhao, Qiao-Ru Liu, Wei-Chun Yang, Song-Tian Li, Xiang Wang

**Affiliations:** a School of Chemistry and Chemical Engineering, Pingdingshan University, Pingdingshan 467000, People’s Republic of China

## Abstract

In each of the two independent mol­ecules in the asymmetric unit of the title compound, [Cu(C_13_H_15_NO_3_)(C_3_H_4_N_2_)], the Cu^II^ atom is four-coordinated by two O atoms and the N atom of the tridentate Schiff base ligand and one N atom from the imidazole ligand in a distorted square-planar geometry. In the crystal structure, mol­ecules are linked by inter­molecular N—H⋯O hydrogen bonds.

## Related literature

For related literature, see: Basu Baul *et al.* (2007 ▶); Casella & Guillotti (1983 ▶); Ganguly *et al.* (2008 ▶); Parekh *et al.* (2006 ▶); Plesch *et al.* (1997 ▶); Usman *et al.* (2003 ▶); Vigato & Tamburini (2004 ▶).
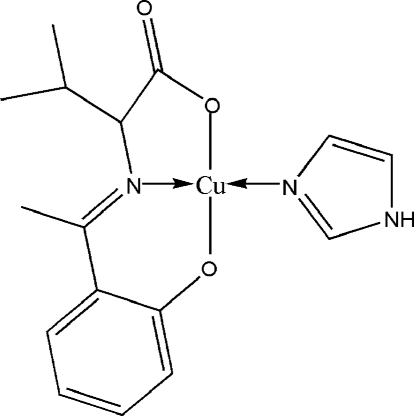


## Experimental

### 

#### Crystal data

[Cu(C_13_H_15_NO_3_)(C_3_H_4_N_2_)]
*M* *_r_* = 364.88
Orthorhombic, 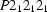

*a* = 12.2025 (13) Å
*b* = 13.5248 (14) Å
*c* = 19.791 (2) Å
*V* = 3266.2 (6) Å^3^
*Z* = 8
Mo *K*α radiation
μ = 1.35 mm^−1^
*T* = 296 (2) K
0.30 × 0.20 × 0.20 mm

#### Data collection

Bruker SMART CCD area-detector diffractometer
Absorption correction: multi-scan (*SADABS*; Sheldrick, 1996 ▶) *T*_min_ = 0.687, *T*_max_ = 0.773
17018 measured reflections
5764 independent reflections
4619 reflections with *I* > 2σ(*I*)
*R*_int_ = 0.031

#### Refinement

*R*[*F* ^2^ > 2σ(*F* ^2^)] = 0.037
*wR*(*F* ^2^) = 0.086
*S* = 1.03
5764 reflections
421 parameters
H-atom parameters constrained
Δρ_max_ = 0.33 e Å^−3^
Δρ_min_ = −0.50 e Å^−3^
Absolute structure: Flack (1983 ▶), 2509 Friedel pairs
Flack parameter: 0.004 (13)

### 

Data collection: *SMART* (Bruker, 2000 ▶); cell refinement: *SAINT* (Bruker, 2000 ▶); data reduction: *SAINT*; program(s) used to solve structure: *SHELXS97* (Sheldrick, 2008 ▶); program(s) used to refine structure: *SHELXL97* (Sheldrick, 2008 ▶); molecular graphics: *SHELXTL* (Sheldrick, 2008 ▶); software used to prepare material for publication: *SHELXTL*.

## Supplementary Material

Crystal structure: contains datablocks global, I. DOI: 10.1107/S1600536808028134/at2627sup1.cif

Structure factors: contains datablocks I. DOI: 10.1107/S1600536808028134/at2627Isup2.hkl

Additional supplementary materials:  crystallographic information; 3D view; checkCIF report

## Figures and Tables

**Table:** 

Cu1—O1	1.867 (3)
Cu1—N1	1.941 (3)
Cu1—O2	1.945 (2)
Cu1—N2	1.972 (3)
Cu2—O4	1.868 (3)
Cu2—N4	1.937 (3)
Cu2—O5	1.949 (3)
Cu2—N5	1.969 (3)

**Table:** 

O1—Cu1—N1	92.94 (13)
O1—Cu1—O2	175.69 (13)
N1—Cu1—O2	85.25 (12)
O1—Cu1—N2	90.51 (12)
N1—Cu1—N2	169.97 (14)
O2—Cu1—N2	91.92 (11)
O4—Cu2—N4	92.79 (13)
O4—Cu2—O5	173.64 (14)
N4—Cu2—O5	84.85 (12)
O4—Cu2—N5	90.96 (12)
N4—Cu2—N5	170.17 (14)
O5—Cu2—N5	92.32 (12)

**Table 2:** Hydrogen-bond geometry (Å, °)

*D*—H⋯*A*	*D*—H	H⋯*A*	*D*⋯*A*	*D*—H⋯*A*
N3—H3*A*⋯O6^i^	0.86	1.91	2.764 (4)	172

Symmetry code: (i) 
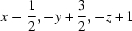
.

**Fig. 1.**
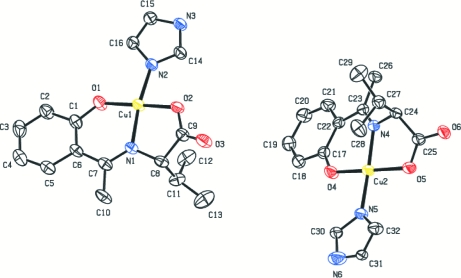
The structure of the title compound (I), showing 30% probability displacement ellipsoids and the atom-numbering scheme. For clarity, H atoms have been omitted.

**Fig. 2.**
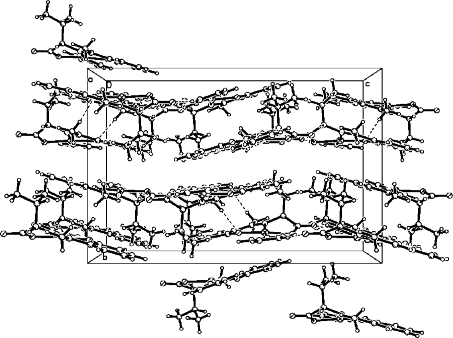
A view of the crystal packing of (I) along the a axis. Hydrogen bonds are shown as dashed lines.

## supplementary crystallographic information

### Comment

In the past decades, significant progress has been achieved in understanding the chemistry of transition metal complexes with Schiff base ligands composed of salicylaldehyde, 2-formylpyridine or their analogues, and α-amino acids (Vigato & Tamburini, 2004; Ganguly *et al.*, 2008; Casella & Guillotti, 1983). A few stuctural studies have been performed on Schiff base complexes derived from 2-hydroxyacetophenone and animo acids (Usman *et al.*, 2003; Basu Baul *et al.*, 2007; Parekh *et al.*, 2006). We report here the crystal structure of the title Cu^II^ complex, (I).

In the title compound (I), the asymmetric unit contains two independent molecules (Fig. 1). The structure consists of discrete monomeric square-planar Cu^II^ complex (Table 1). The four basal positions are occupied by three donor atoms from the tridentate Schiff base ligand, which furnishes an ONO donor set, with the fourth position occupied by one N atom from the imidazole ligand. In the molecules with Cu1 and Cu2, the nitrogen heterocycles are planar and they form the angles of 4.2 (2) and 6.0 (2)° with the C1—C6 and C17–C32 rings, respectively.

The crystal structure is stabilized by intermolecular N—H···O hydrogen bonds (Fig. 2 and Table 2), which the H atom attached to N3 is hydrogen-bonded to the neighboring carboxylate oxygen O6.

### Experimental

The title compound was synthesized as described in the literature (Plesch *et al.*, 1997). To *L*-valine (1.00 mmol) and potassium hydroxide (1.00 mmol) in 10 ml of methanol was added 2-hydroxyacetophenone (1.00 mmol in 10 ml of methanol) dropwise. The yellow solution was stirred for 2.0 h at 333 K. The resultant mixture was added dropwise to copper(II) acetate monohydrate (1.00 mmol) and imidazole (1.00 mmol) in an aqueous methanolic solution (20 ml, 1:1 *v*/*v*), and heated with stirring for 2.0 h at 333 K. The dark blue solution was filtered and left for several days, dark blue crystals had formed that were filtered off, washed with water, and dried under vacuum.

### Refinement

All H atoms were positioned geometrically and refined as riding atoms, with C—H = 0.93 or 0.98 Å (CH) and *U*_iso_(H) = 1.2*U*_eq_(C), with C—H = 0.96 Å (CH_3_) and *U*_iso_(H) = 1.5*U*_eq_(C), and with N—H = 0.86 Å (NH) and *U*_iso_(H) = 1.2*U*_eq_(N).

### Crystal data

**Table:**

[Cu(C_13_H_15_NO_3_)(C_3_H_4_N_2_)]	*F*(000) = 1512
*M**_r_* = 364.88	*D*_x_ = 1.484 Mg m^−^^3^
Orthorhombic, *P*2_1_2_1_2_1_	Mo *K*α radiation, λ = 0.71073 Å
Hall symbol: P 2ac 2ab	Cell parameters from 4789 reflections
*a* = 12.2025 (13) Å	θ = 2.3–23.6°
*b* = 13.5248 (14) Å	µ = 1.36 mm^−^^1^
*c* = 19.791 (2) Å	*T* = 296 K
*V* = 3266.2 (6) Å^3^	Block, dark blue
*Z* = 8	0.30 × 0.20 × 0.20 mm

### Data collection

**Table:**

Bruker SMART CCD area-detector diffractometer	5764 independent reflections
Radiation source: fine-focus sealed tube	4619 reflections with *I* > 2σ(*I*)
graphite	*R*_int_ = 0.031
φ and ω scans	θ_max_ = 25.1°, θ_min_ = 2.0°
Absorption correction: multi-scan (SADABS; Sheldrick, 1996)	*h* = −14→14
*T*_min_ = 0.687, *T*_max_ = 0.773	*k* = −14→16
17018 measured reflections	*l* = −23→21

### Refinement

**Table:**

Refinement on *F*^2^	Secondary atom site location: difference Fourier map
Least-squares matrix: full	Hydrogen site location: inferred from neighbouring sites
*R*[*F*^2^ > 2σ(*F*^2^)] = 0.037	H-atom parameters constrained
*wR*(*F*^2^) = 0.086	*w* = 1/[σ^2^(*F*_o_^2^) + (0.0297*P*)^2^ + 1.2486*P*] where *P* = (*F*_o_^2^ + 2*F*_c_^2^)/3
*S* = 1.03	(Δ/σ)_max_ = 0.001
5764 reflections	Δρ_max_ = 0.33 e Å^−^^3^
421 parameters	Δρ_min_ = −0.50 e Å^−^^3^
0 restraints	Absolute structure: Flack (1983), 2509 Freidel pairs
Primary atom site location: structure-invariant direct methods	Flack parameter: 0.004 (13)

### Special details

**Table:**

Geometry. All e.s.d.'s (except the e.s.d. in the dihedral angle between two l.s. planes) are estimated using the full covariance matrix. The cell e.s.d.'s are taken into account individually in the estimation of e.s.d.'s in distances, angles and torsion angles; correlations between e.s.d.'s in cell parameters are only used when they are defined by crystal symmetry. An approximate (isotropic) treatment of cell e.s.d.'s is used for estimating e.s.d.'s involving l.s. planes.
Refinement. Refinement of *F*^2^ against ALL reflections. The weighted *R*-factor *wR* and goodness of fit *S* are based on *F*^2^, conventional *R*-factors *R* are based on *F*, with *F* set to zero for negative *F*^2^. The threshold expression of *F*^2^ > σ(*F*^2^) is used only for calculating *R*-factors(gt) *etc*. and is not relevant to the choice of reflections for refinement. *R*-factors based on *F*^2^ are statistically about twice as large as those based on *F*, and *R*- factors based on ALL data will be even larger.

### Fractional atomic coordinates and isotropic or equivalent isotropic displacement parameters (Å^2^)

**Table:**

	*x*	*y*	*z*	*U*_iso_*/*U*_eq_	
Cu1	0.52180 (4)	0.63527 (4)	0.08491 (2)	0.04826 (14)	
Cu2	0.65002 (3)	0.85812 (4)	0.60823 (2)	0.04751 (14)	
C1	0.6063 (3)	0.6039 (3)	−0.0474 (2)	0.0532 (11)	
C2	0.5832 (4)	0.5745 (4)	−0.1145 (2)	0.0655 (13)	
H2	0.5106	0.5699	−0.1284	0.079*	
C3	0.6650 (5)	0.5528 (4)	−0.1596 (2)	0.0745 (14)	
H3	0.6474	0.5333	−0.2033	0.089*	
C4	0.7750 (4)	0.5597 (4)	−0.1401 (3)	0.0738 (15)	
H4	0.8310	0.5448	−0.1703	0.089*	
C5	0.7979 (4)	0.5886 (3)	−0.0761 (3)	0.0624 (12)	
H5	0.8711	0.5944	−0.0637	0.075*	
C6	0.7169 (3)	0.6106 (3)	−0.0269 (2)	0.0506 (11)	
C7	0.7512 (3)	0.6428 (3)	0.0404 (2)	0.0515 (10)	
C8	0.7081 (3)	0.6986 (3)	0.1538 (2)	0.0490 (10)	
H8	0.7813	0.6752	0.1665	0.059*	
C9	0.6246 (3)	0.6621 (3)	0.2057 (2)	0.0503 (11)	
C10	0.8720 (3)	0.6564 (4)	0.0537 (2)	0.0719 (15)	
H10A	0.9015	0.7031	0.0221	0.108*	
H10B	0.9088	0.5941	0.0487	0.108*	
H10C	0.8824	0.6805	0.0989	0.108*	
C11	0.7088 (4)	0.8124 (3)	0.1499 (2)	0.0636 (13)	
H11	0.7550	0.8299	0.1112	0.076*	
C12	0.5957 (4)	0.8557 (4)	0.1357 (3)	0.0809 (15)	
H12A	0.5476	0.8413	0.1728	0.121*	
H12B	0.5667	0.8270	0.0951	0.121*	
H12C	0.6015	0.9260	0.1302	0.121*	
C13	0.7593 (5)	0.8599 (5)	0.2100 (3)	0.1054 (19)	
H13A	0.7127	0.8503	0.2486	0.158*	
H13B	0.7681	0.9294	0.2017	0.158*	
H13C	0.8296	0.8306	0.2185	0.158*	
C14	0.2976 (3)	0.6635 (3)	0.1369 (2)	0.0558 (12)	
H14	0.3233	0.6875	0.1780	0.067*	
C15	0.1879 (3)	0.6175 (3)	0.0578 (2)	0.0567 (12)	
H15	0.1251	0.6030	0.0331	0.068*	
C16	0.2909 (3)	0.6085 (3)	0.0364 (2)	0.0524 (11)	
H16	0.3120	0.5863	−0.0061	0.063*	
C17	0.5693 (3)	0.8710 (3)	0.47462 (19)	0.0457 (9)	
C18	0.5908 (3)	0.8916 (3)	0.4066 (2)	0.0539 (11)	
H18	0.6633	0.8948	0.3923	0.065*	
C19	0.5093 (4)	0.9074 (3)	0.3601 (2)	0.0606 (12)	
H19	0.5269	0.9213	0.3154	0.073*	
C20	0.4009 (4)	0.9024 (3)	0.3801 (3)	0.0625 (13)	
H20	0.3449	0.9150	0.3494	0.075*	
C21	0.3770 (3)	0.8788 (3)	0.4455 (2)	0.0584 (12)	
H21	0.3037	0.8742	0.4579	0.070*	
C22	0.4572 (3)	0.8610 (3)	0.49521 (18)	0.0433 (9)	
C23	0.4247 (3)	0.8301 (3)	0.5626 (2)	0.0444 (10)	
C24	0.4742 (3)	0.7757 (3)	0.67720 (18)	0.0465 (9)	
H24	0.3968	0.7869	0.6881	0.056*	
C25	0.5459 (3)	0.8289 (3)	0.7284 (2)	0.0537 (12)	
C26	0.3039 (3)	0.8159 (3)	0.5762 (2)	0.0554 (11)	
H26A	0.2935	0.7965	0.6225	0.083*	
H26B	0.2756	0.7653	0.5470	0.083*	
H26C	0.2657	0.8767	0.5679	0.083*	
C27	0.4998 (3)	0.6645 (3)	0.6814 (2)	0.0556 (11)	
H27	0.4895	0.6449	0.7286	0.067*	
C28	0.6189 (4)	0.6388 (4)	0.6627 (2)	0.0792 (14)	
H28A	0.6310	0.5694	0.6695	0.119*	
H28B	0.6681	0.6758	0.6909	0.119*	
H28C	0.6319	0.6552	0.6162	0.119*	
C29	0.4234 (4)	0.6023 (4)	0.6399 (3)	0.0898 (18)	
H29A	0.3491	0.6161	0.6527	0.135*	
H29B	0.4390	0.5336	0.6475	0.135*	
H29C	0.4333	0.6174	0.5929	0.135*	
C30	0.8785 (3)	0.8880 (3)	0.5586 (2)	0.0519 (11)	
H30	0.8581	0.8966	0.5137	0.062*	
C31	0.9781 (3)	0.8710 (3)	0.64947 (17)	0.0363 (8)	
H31	1.0370	0.8654	0.6791	0.044*	
C32	0.8746 (3)	0.8686 (4)	0.6653 (2)	0.0588 (11)	
H32	0.8492	0.8608	0.7093	0.071*	
N1	0.6788 (2)	0.6579 (2)	0.08787 (16)	0.0469 (8)	
N2	0.3602 (2)	0.6369 (3)	0.08634 (15)	0.0482 (7)	
N3	0.1914 (2)	0.6513 (3)	0.12130 (17)	0.0559 (9)	
H3A	0.1362	0.6630	0.1471	0.067*	
N4	0.4983 (2)	0.8161 (2)	0.60987 (17)	0.0437 (7)	
N5	0.8097 (2)	0.8782 (2)	0.61290 (16)	0.0478 (8)	
N6	0.9835 (3)	0.8827 (3)	0.5834 (3)	0.0933 (16)	
H6	1.0426	0.8865	0.5599	0.112*	
O1	0.5208 (2)	0.6222 (2)	−0.00899 (13)	0.0623 (8)	
O2	0.52949 (19)	0.6399 (2)	0.18300 (12)	0.0534 (7)	
O3	0.6509 (2)	0.6600 (3)	0.26604 (14)	0.0701 (10)	
O4	0.65434 (19)	0.8605 (3)	0.51391 (13)	0.0631 (8)	
O5	0.6344 (2)	0.8681 (3)	0.70602 (13)	0.0640 (8)	
O6	0.5198 (2)	0.8268 (3)	0.78876 (14)	0.0691 (9)	

### Atomic displacement parameters (Å^2^)

**Table:**

	*U*^11^	*U*^22^	*U*^33^	*U*^12^	*U*^13^	*U*^23^
Cu1	0.0364 (2)	0.0606 (3)	0.0478 (3)	0.0005 (3)	0.0044 (2)	0.0034 (3)
Cu2	0.0345 (2)	0.0625 (3)	0.0455 (3)	−0.0071 (2)	−0.0017 (2)	−0.0016 (3)
C1	0.056 (2)	0.056 (3)	0.048 (3)	0.008 (2)	0.010 (2)	0.009 (2)
C2	0.060 (3)	0.084 (3)	0.053 (3)	0.004 (2)	0.005 (2)	0.008 (3)
C3	0.099 (4)	0.077 (3)	0.047 (3)	0.001 (3)	0.013 (3)	0.003 (2)
C4	0.075 (3)	0.075 (4)	0.071 (4)	0.011 (3)	0.028 (3)	0.005 (3)
C5	0.051 (2)	0.064 (3)	0.072 (3)	0.002 (2)	0.015 (2)	0.007 (3)
C6	0.045 (2)	0.051 (3)	0.055 (3)	0.0040 (19)	0.0146 (19)	0.0125 (19)
C7	0.043 (2)	0.051 (3)	0.060 (3)	0.007 (2)	0.0099 (19)	0.012 (3)
C8	0.0331 (19)	0.061 (3)	0.053 (3)	0.0006 (19)	0.0002 (18)	0.013 (2)
C9	0.042 (2)	0.059 (3)	0.050 (3)	0.0055 (19)	0.0032 (19)	0.006 (2)
C10	0.037 (2)	0.109 (4)	0.069 (3)	0.006 (3)	0.015 (2)	0.016 (3)
C11	0.055 (3)	0.063 (3)	0.072 (3)	−0.011 (2)	−0.013 (2)	0.003 (2)
C12	0.079 (3)	0.057 (3)	0.107 (4)	0.008 (3)	−0.033 (3)	−0.003 (3)
C13	0.117 (5)	0.098 (4)	0.102 (4)	−0.018 (4)	−0.044 (4)	0.000 (4)
C14	0.041 (2)	0.078 (3)	0.049 (2)	0.001 (2)	0.0043 (19)	−0.005 (2)
C15	0.043 (2)	0.080 (3)	0.047 (2)	0.001 (2)	−0.0065 (19)	−0.002 (2)
C16	0.048 (2)	0.069 (3)	0.040 (2)	0.000 (2)	−0.0019 (19)	−0.002 (2)
C17	0.043 (2)	0.049 (2)	0.046 (2)	0.003 (2)	−0.0036 (18)	−0.004 (2)
C18	0.046 (2)	0.069 (3)	0.046 (3)	0.002 (2)	−0.0026 (19)	0.000 (2)
C19	0.066 (3)	0.064 (3)	0.051 (3)	0.001 (2)	0.000 (2)	0.004 (2)
C20	0.056 (3)	0.076 (3)	0.056 (3)	0.000 (2)	−0.014 (2)	0.011 (2)
C21	0.041 (2)	0.071 (3)	0.064 (3)	0.000 (2)	−0.006 (2)	0.001 (2)
C22	0.0382 (19)	0.048 (2)	0.044 (2)	−0.001 (2)	−0.0069 (16)	−0.003 (2)
C23	0.0374 (19)	0.047 (3)	0.049 (2)	−0.0028 (17)	0.0002 (18)	−0.0089 (18)
C24	0.0325 (18)	0.062 (3)	0.045 (2)	−0.0064 (19)	0.0031 (18)	−0.0033 (19)
C25	0.034 (2)	0.076 (3)	0.050 (3)	−0.003 (2)	−0.0044 (19)	−0.005 (2)
C26	0.034 (2)	0.077 (3)	0.054 (3)	−0.003 (2)	−0.0013 (19)	−0.006 (2)
C27	0.054 (2)	0.065 (3)	0.048 (2)	−0.004 (2)	0.0015 (19)	0.003 (2)
C28	0.074 (3)	0.082 (3)	0.081 (3)	0.021 (3)	0.018 (3)	0.012 (3)
C29	0.105 (4)	0.074 (4)	0.091 (4)	−0.012 (3)	−0.018 (3)	−0.003 (3)
C30	0.041 (2)	0.068 (3)	0.047 (2)	−0.009 (2)	−0.0002 (18)	−0.006 (2)
C31	0.0283 (17)	0.053 (2)	0.0273 (18)	−0.003 (2)	−0.0090 (15)	0.0052 (19)
C32	0.052 (2)	0.075 (3)	0.049 (2)	−0.013 (3)	−0.005 (2)	0.016 (3)
N1	0.0393 (17)	0.054 (2)	0.0472 (19)	−0.0001 (15)	0.0052 (15)	0.0091 (17)
N2	0.0408 (16)	0.060 (2)	0.0439 (18)	−0.0004 (18)	0.0032 (14)	−0.001 (2)
N3	0.0388 (17)	0.076 (3)	0.053 (2)	0.0006 (19)	0.0066 (15)	−0.001 (2)
N4	0.0353 (16)	0.0518 (19)	0.0440 (18)	−0.0038 (14)	−0.0009 (14)	−0.0054 (15)
N5	0.0383 (16)	0.060 (2)	0.0451 (18)	−0.0067 (17)	0.0001 (15)	0.0047 (18)
N6	0.047 (2)	0.083 (3)	0.149 (5)	−0.002 (2)	0.002 (3)	−0.024 (3)
O1	0.0461 (15)	0.095 (2)	0.0457 (16)	0.0086 (19)	0.0045 (13)	0.0038 (16)
O2	0.0373 (13)	0.0749 (18)	0.0479 (15)	−0.0075 (18)	0.0039 (12)	0.0035 (16)
O3	0.0458 (15)	0.115 (3)	0.0492 (18)	−0.0072 (18)	−0.0004 (14)	0.0161 (18)
O4	0.0356 (13)	0.107 (2)	0.0465 (16)	−0.003 (2)	−0.0004 (12)	−0.0003 (18)
O5	0.0450 (15)	0.096 (2)	0.0507 (17)	−0.019 (2)	−0.0016 (13)	−0.0106 (18)
O6	0.0445 (15)	0.121 (3)	0.0417 (18)	−0.0093 (17)	0.0043 (14)	−0.0130 (16)

### Geometric parameters (Å, °)

**Table:**

Cu1—O1	1.867 (3)	C15—H15	0.9300
Cu1—N1	1.941 (3)	C16—N2	1.356 (5)
Cu1—O2	1.945 (2)	C16—H16	0.9300
Cu1—N2	1.972 (3)	C17—O4	1.304 (4)
Cu2—O4	1.868 (3)	C17—C18	1.399 (5)
Cu2—N4	1.937 (3)	C17—C22	1.433 (5)
Cu2—O5	1.949 (3)	C18—C19	1.371 (5)
Cu2—N5	1.969 (3)	C18—H18	0.9300
C1—O1	1.314 (5)	C19—C20	1.383 (6)
C1—C6	1.412 (6)	C19—H19	0.9300
C1—C2	1.415 (6)	C20—C21	1.363 (6)
C2—C3	1.371 (6)	C20—H20	0.9300
C2—H2	0.9300	C21—C22	1.409 (5)
C3—C4	1.399 (7)	C21—H21	0.9300
C3—H3	0.9300	C22—C23	1.453 (5)
C4—C5	1.355 (6)	C23—N4	1.311 (5)
C4—H4	0.9300	C23—C26	1.511 (5)
C5—C6	1.419 (6)	C24—N4	1.470 (5)
C5—H5	0.9300	C24—C25	1.520 (5)
C6—C7	1.464 (6)	C24—C27	1.539 (6)
C7—N1	1.305 (4)	C24—H24	0.9800
C7—C10	1.508 (5)	C25—O6	1.236 (5)
C8—N1	1.461 (5)	C25—O5	1.282 (4)
C8—C9	1.528 (5)	C26—H26A	0.9600
C8—C11	1.541 (6)	C26—H26B	0.9600
C8—H8	0.9800	C26—H26C	0.9600
C9—O3	1.237 (5)	C27—C29	1.500 (6)
C9—O2	1.281 (4)	C27—C28	1.539 (6)
C10—H10A	0.9600	C27—H27	0.9800
C10—H10B	0.9600	C28—H28A	0.9600
C10—H10C	0.9600	C28—H28B	0.9600
C11—C13	1.484 (6)	C28—H28C	0.9600
C11—C12	1.526 (6)	C29—H29A	0.9600
C11—H11	0.9800	C29—H29B	0.9600
C12—H12A	0.9600	C29—H29C	0.9600
C12—H12B	0.9600	C30—N5	1.370 (5)
C12—H12C	0.9600	C30—N6	1.375 (5)
C13—H13A	0.9600	C30—H30	0.9300
C13—H13B	0.9600	C31—C32	1.302 (5)
C13—H13C	0.9600	C31—N6	1.319 (5)
C14—N2	1.310 (5)	C31—H31	0.9300
C14—N3	1.343 (5)	C32—N5	1.311 (5)
C14—H14	0.9300	C32—H32	0.9300
C15—C16	1.332 (5)	N3—H3A	0.8600
C15—N3	1.338 (5)	N6—H6	0.8600
			
O1—Cu1—N1	92.94 (13)	C19—C18—H18	118.6
O1—Cu1—O2	175.69 (13)	C17—C18—H18	118.6
N1—Cu1—O2	85.25 (12)	C18—C19—C20	119.6 (4)
O1—Cu1—N2	90.51 (12)	C18—C19—H19	120.2
N1—Cu1—N2	169.97 (14)	C20—C19—H19	120.2
O2—Cu1—N2	91.92 (11)	C21—C20—C19	119.2 (4)
O4—Cu2—N4	92.79 (13)	C21—C20—H20	120.4
O4—Cu2—O5	173.64 (14)	C19—C20—H20	120.4
N4—Cu2—O5	84.85 (12)	C20—C21—C22	123.7 (4)
O4—Cu2—N5	90.96 (12)	C20—C21—H21	118.2
N4—Cu2—N5	170.17 (14)	C22—C21—H21	118.2
O5—Cu2—N5	92.32 (12)	C21—C22—C17	116.6 (3)
O1—C1—C6	125.5 (4)	C21—C22—C23	120.1 (3)
O1—C1—C2	116.0 (4)	C17—C22—C23	123.3 (3)
C6—C1—C2	118.5 (4)	N4—C23—C22	120.7 (3)
C3—C2—C1	121.8 (5)	N4—C23—C26	121.5 (4)
C3—C2—H2	119.1	C22—C23—C26	117.8 (3)
C1—C2—H2	119.1	N4—C24—C25	108.3 (3)
C2—C3—C4	120.3 (5)	N4—C24—C27	111.8 (3)
C2—C3—H3	119.9	C25—C24—C27	108.0 (3)
C4—C3—H3	119.9	N4—C24—H24	109.6
C5—C4—C3	118.4 (5)	C25—C24—H24	109.6
C5—C4—H4	120.8	C27—C24—H24	109.6
C3—C4—H4	120.8	O6—C25—O5	124.1 (4)
C4—C5—C6	123.9 (5)	O6—C25—C24	119.0 (4)
C4—C5—H5	118.0	O5—C25—C24	116.7 (4)
C6—C5—H5	118.0	C23—C26—H26A	109.5
C1—C6—C5	117.1 (4)	C23—C26—H26B	109.5
C1—C6—C7	123.6 (4)	H26A—C26—H26B	109.5
C5—C6—C7	119.2 (4)	C23—C26—H26C	109.5
N1—C7—C6	120.6 (3)	H26A—C26—H26C	109.5
N1—C7—C10	121.1 (4)	H26B—C26—H26C	109.5
C6—C7—C10	118.3 (3)	C29—C27—C28	109.2 (4)
N1—C8—C9	108.3 (3)	C29—C27—C24	113.1 (4)
N1—C8—C11	109.5 (3)	C28—C27—C24	113.6 (4)
C9—C8—C11	111.1 (4)	C29—C27—H27	106.8
N1—C8—H8	109.3	C28—C27—H27	106.8
C9—C8—H8	109.3	C24—C27—H27	106.8
C11—C8—H8	109.3	C27—C28—H28A	109.5
O3—C9—O2	124.6 (4)	C27—C28—H28B	109.5
O3—C9—C8	118.9 (4)	H28A—C28—H28B	109.5
O2—C9—C8	116.4 (4)	C27—C28—H28C	109.5
C7—C10—H10A	109.5	H28A—C28—H28C	109.5
C7—C10—H10B	109.5	H28B—C28—H28C	109.5
H10A—C10—H10B	109.5	C27—C29—H29A	109.5
C7—C10—H10C	109.5	H29A—C29—H29C	109.5
H10A—C10—H10C	109.5	H29B—C29—H29C	109.5
H10B—C10—H10C	109.5	N5—C30—N6	106.6 (4)
C13—C11—C12	111.0 (4)	N5—C30—H30	126.7
C13—C11—C8	113.2 (4)	N6—C30—H30	126.7
C12—C11—C8	112.8 (4)	C32—C31—N6	106.9 (3)
C13—C11—H11	106.5	C32—C31—H31	126.6
C12—C11—H11	106.5	N6—C31—H31	126.6
C8—C11—H11	106.5	C31—C32—N5	113.2 (4)
C11—C12—H12A	109.5	C31—C32—H32	123.4
C11—C12—H12B	109.5	N5—C32—H32	123.4
H12A—C12—H12B	109.5	C7—N1—C8	122.4 (3)
C11—C12—H12C	109.5	C7—N1—Cu1	128.4 (3)
H12A—C12—H12C	109.5	C8—N1—Cu1	109.1 (2)
H12B—C12—H12C	109.5	C14—N2—C16	105.8 (3)
C11—C13—H13A	109.5	C14—N2—Cu1	126.6 (3)
C11—C13—H13B	109.5	C16—N2—Cu1	127.6 (3)
H13A—C13—H13B	109.5	C15—N3—C14	106.8 (3)
C11—C13—H13C	109.5	C15—N3—H3A	126.6
H13A—C13—H13C	109.5	C14—N3—H3A	126.6
H13B—C13—H13C	109.5	C23—N4—C24	124.3 (3)
N2—C14—N3	110.7 (4)	C23—N4—Cu2	126.9 (3)
N2—C14—H14	124.7	C24—N4—Cu2	108.4 (2)
N3—C14—H14	124.7	C32—N5—C30	105.1 (3)
C16—C15—N3	107.5 (4)	C32—N5—Cu2	128.4 (3)
C16—C15—H15	126.3	C30—N5—Cu2	125.6 (3)
N3—C15—H15	126.3	C31—N6—C30	108.3 (4)
C15—C16—N2	109.3 (4)	C31—N6—H6	125.9
C15—C16—H16	125.3	C30—N6—H6	125.9
N2—C16—H16	125.3	C1—O1—Cu1	126.0 (3)
O4—C17—C18	116.5 (3)	C9—O2—Cu1	113.6 (2)
O4—C17—C22	125.4 (3)	C17—O4—Cu2	125.1 (2)
C18—C17—C22	118.1 (3)	C25—O5—Cu2	113.4 (2)
C19—C18—C17	122.7 (4)		

### Hydrogen-bond geometry (Å, °)

**Table:**

*D*—H···*A*	*D*—H	H···*A*	*D*···*A*	*D*—H···*A*
N3—H3A···O6^i^	0.86	1.91	2.764 (4)	172.

Symmetry codes: (i) *x*−1/2, −*y*+3/2, −*z*+1.
